# ITGA1 is a pre-malignant biomarker that promotes therapy resistance and metastatic potential in pancreatic cancer

**DOI:** 10.1038/s41598-017-09946-z

**Published:** 2017-08-30

**Authors:** Armen Gharibi, Sa La Kim, Justin Molnar, Daniel Brambilla, Yvess Adamian, Malachia Hoover, Julie Hong, Joy Lin, Laurelin Wolfenden, Jonathan A. Kelber

**Affiliations:** Department of Biology, California State Univeristy Northridge, Northridge, California USA

## Abstract

Pancreatic ductal adenocarcinoma (PDAC) has single-digit 5-year survival rates at <7%. There is a dire need to improve pre-malignant detection methods and identify new therapeutic targets for abrogating PDAC progression. To this end, we mined our previously published pseudopodium-enriched (PDE) protein/phosphoprotein datasets to identify novel PDAC-specific biomarkers and/or therapeutic targets. We discovered that integrin alpha 1 (ITGA1) is frequently upregulated in pancreatic cancers and associated precursor lesions. Expression of ITGA1-specific collagens within the pancreatic cancer microenvironment significantly correlates with indicators of poor patient prognosis, and depleting ITGA1 from PDAC cells revealed that it is required for collagen-induced tumorigenic potential. Notably, collagen/ITGA1 signaling promotes the survival of ALDH1-positive stem-like cells and cooperates with TGFβ to drive gemcitabine resistance. Finally, we report that ITGA1 is required for TGFβ/collagen-induced EMT and metastasis. Our data suggest that ITGA1 is a new diagnostic biomarker and target that can be leveraged to improve patient outcomes.

## Introduction

While pancreatic cancer (pancreatic ductal adenocarcinoma or PDAC) research efforts and the tools with which to study this malignancy have increased drastically over the past few decades, the 5-year survival rate remains at only 7% and the median survival from the time of diagnosis is less than 12 months^1–3^. This high mortality among patients is partially due to limited diagnostic methods for identifying non-invasive/-metastatic forms of this disease. In this regard, distant metastases have been reported as being observed late in relation to the predicted genetic evolution of pancreatic cancer^4^, suggesting that at least a 10-year window exists during which diagnoses may be made to improve outcomes. Still, early diagnosis of pancreatic cancer prior to systemic metastasis only increases patient survival to approximately 20%, further pointing to a critical need for diagnostic tools targeting pre-malignant pancreatic tissue (e.g., pancreatic intraepithelial neosplasms or PanINs). Recent work has underscored this need, showing that epithelial to mesenchymal transition (EMT) and systemic dissemination of pancreatic tumor cells occurs well before primary tumors are detectable within the pancreas^5, 6^.

An additional complicating factor that contributes to the dismal patient prognosis in PDAC is its therapy refractory nature. This is due primarily to the heterogeneity of the highly desmoplastic tumor microenvironment^7^. To combat this cellular and molecular complexity, and improve therapy responses at both primary and metastatic tumor sites, it has become apparent that future therapeutic approaches will need to target the interaction between rare stem-like cancer cells and their tumor-protective environment^8–10^.

In this regard, we mined our previously published peudopodium proteomic data for proteins frequently upregulated in PDAC and that regulate cell-extracellular interactions. Integrins are the primary transmembrane receptors that transmit intracellular signals when they bind to their respective extracellular matrix (ECM) proteins. Integrins function as heterodimers, in which the active integrin complex consists of alpha and beta subunits^11^. We subsequently identified integrin alpha 1 (ITGA1) as a pseudopodium-enriched protein frequently upregulated in PDAC. Therefore, we hypothesized that its subcellular localization to the cell surface membrane of pseudopodia may make it an ideal target for diagnostic and therapeutic interventions in this malignancy. Notably, we discovered that ITGA1-binding collagens are most frequently upregulated in PDAC and statistically associate with indicators of poor patient prognosis. We also report that ITGA1 is upregulated in PanIN tissue in the human pancreas. Our *in vitro* studies provide compelling evidence that ITGA1 is necessary for survival of stem-like PDAC cells and gemcitabine resistance in these cell populations; migration, attachment and spreading of PDAC cells; and TGFβ/collagen-induced EMT. *In vivo*, we also demonstrate a requirement for ITGA1 during PDAC metastasis to lung, brain and liver tissues. This work provides rationale for using ITGA1 as a diagnostic biomarker and therapeutic target to improve patient survival.

## Results

### Pseudopodium-enriched proteins are viable cancer biomarkers

Previous work has established methods to isolate protruding pseudopodia from migrating cells for downstream proteomic and phosphoproteomic analyses^12–14^. Importantly, we previously utilized these methods for the purpose of identifying the novel non-receptor cytoskeleton-associated tyrosine kinase PEAK1 (pseudopodium-enriched atypical kinase one)^15^. More recent work from our group has demonstrated a clear role for PEAK1 in the initiation and progression of both breast and pancreatic malignancies^16–19^. Since pseudopodia are comprised of proteins known to regulate cancer cell interactions with their microenvironment and overall disease progression^20, 21^, we further reasoned that pseudopodia-enriched (PDE) proteins may generally be good candidate biomarkers of cancer initiation, progression or therapy response. We first sought to identify novel biomarkers within pancreatic ductal adenocarcinoma (PDAC) as this cancer diagnosis has a very poor prognosis. We cross-referenced the top 100 PDE proteins (Supplemental Table 1) from our previously published dataset^15^ against 29 gene expression studies of pancreatic cancer patient tissue using Oncomine. The oncomine thresholds were set to identify genes significantly upregulated by more than 1.5 fold in the tumor tissue with a statistical significance of p < 0.05 (Supplemental Fig. 1A). Using these parameters, we report here that 37 percent of the top PDE proteins are upregulated at the transcript level in pancreatic cancer (Supplemental Fig. 1B). To evaluate the significance of these findings we performed hierarchical clustering of the overexpression frequency for these proteins across multiple malignancy types (Supplemental Fig. 1B), demonstrating that a significant proportion of these genes are also frequently upregulated in other cancers and may be functionally related to each other in these other tumor types. Interestingly, while integrin alpha one (ITGA1) is frequently upregulated in PDAC, we also note that there are malignancies in which ITGA1 is more frequently overexpressed (e.g., Melanoma, Prostate, Bladder, Liver and Myeloma). Thus, it may be relevant to characterize ITGA1 function further in these other cancer types. Notably, ITGA1 has recently been associated with an invasive/metastatic phenotype in hepatocellular and prostate cancers^22–24^.

Using Cytoscape, we evaluated the interconnectedness of this 37 gene set list (Supplemental Fig. 1C) using the Agilent Literature Search plugin and a subnetwork of this larger interactome focusing on the sole extracellular matrix protein receptor or integrin, ITGA1, that was in our 37 gene set list (Supplemental Fig. 1C and D). Finally, we analyzed the gene ontologies of these 37 PDE proteins that are upregulated in PDAC to evaluate the enrichment of associated biological processes – the top 12 are shown in Supplemental Fig. 1E. Of note, many of the integrin- and small GTPase-mediated signaling pathway components are known to have roles in these other biological processes.

### ITGA1 is upregulated in PanIN and PDAC tissues and associates with indicators of poor prognosis

**Figure 1:**
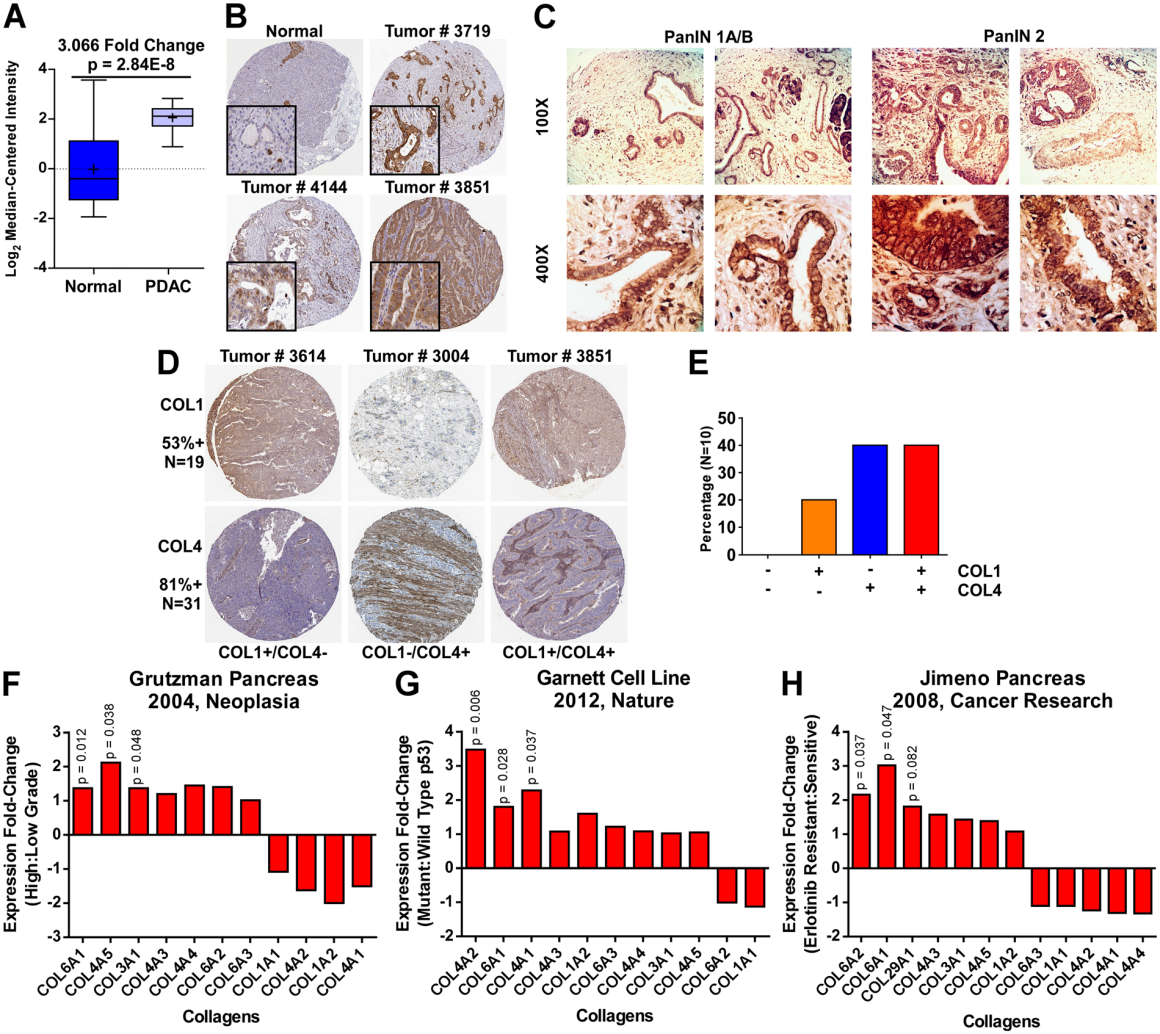
ITGA1 is upregulated in PanIN and PDAC tissues and associates with indicators of poor prognosis. (**A**) ITGA1 expression in normal and malignant pancreatic tissue sampled from 38 patients using Oncomine. (**B**) Immunohistochemistry (IHC) staining for ITGA1 in normal and tumor pancreatic tissue from a Human Protein Atlas Portal. (**C**) IHC staining for ITGA1 in pre-malignant PanIN lesions in human patient samples. (**D**) Immunohistochemistry staining for COL1 and COL4 in tumor pancreatic tissue from a Human Protein Atlas. (**E**) Quantified data from a Human Protein Atlas indicating the percentages of PDAC patients with elevated COL1 and/or COL4 levels. (**F**–**H**) Patient data from Oncomine associating expression of collagen types with tumor grade (**F**), p53 mutation status (**G**) and erlotinib resistance (**H**).

As discussed above, ITGA1 is one of the 37 top PDE proteins significantly upregulated in PDAC (Fig. 1A). We further analyzed ITGA1 expression at the protein level in the normal pancreas and PDAC tissue using data available in the Human Protein Atlas repository^25^. Notably, normal pancreatic ductal epithelial and acinar cells stain negative for ITGA1, while tumor tissue is highly positive across multiple patients, predominantly in the epithelial compartment (Fig. 1B) – high ITGA1 expression is detected in approximately 42% of patient samples. In an effort to develop novel methods to detect PDAC early and improve patient prognosis, researchers have characterized novel proteins that are found to circulate within the blood of PDAC patients and which associate with the presence of pancreatic intraepithelial neoplasms (PanINs)^26^. Together with recent reports that epithelial-mesenchymal transition (EMT) and dissemination of cells from pre-malignant pancreatic tissue can precede tumor formation^5^, a clear rational exists for identifying novel cell surface biomarkers that are upregulated in PanIN tissue. We performed immunohistochemistry (IHC) for ITGA1 on an array of patient pancreatic tissue, observing prominent staining in PanIN 1 A/B and PanIN 2 lesions across all 10 patient samples (Fig. 1C).

A hallmark of pancreatic cancer is desmoplasia (i.e., stromal cell/tissue infiltration into and around the otherwise predominantly epithelial tumor cells)^3, 27^. Collagens are a primary component of the PDAC stromal microenvironment, and ITGB1 (the key beta integrin subunit for collagen binding) has been previously characterized to play an important role during PDAC progression^28, 29^. While ITGA2 and ITGA11 are also upregulated in pancreatic cancer (data available via Oncomine) and can mediate cell-surface binding of various collagen types, neither of these alternate alpha integrins were PDE proteins suggesting that they may play a more context-dependent role in pancreatic cancer^15^. To further establish rationale for investigating ITGA1 function in PDAC, we analyzed the protein levels of collagens that utilize ITGA1 (type 4 collagens) and ITGA2 (type 1 collagens) in PDAC patient samples by IHC^25^. As shown in Fig. 1D, 53% of all PDAC tumors available in the Human Protein Atlas that were stained for type 1 collagens were positive for type 1 collagens (N = 19), while 81% of banked PDAC samples that were stained for type 4 collagens were positive for type 4 collagens (N = 31). Furthermore, of the 10 patient samples that were stained for both type 1 and type 4 collagens, 80% of them stained positively for collagens that bind to ITGA1 (Fig. 1E). Finally, we evaluated a broader panel of collagen types in their predictive power of tumor grade, p53 mutation status and erlotinib response status using Oncomine^30–32^. Grutzman and colleagues microdissected normal and malignant pancreatic tissue from patients on which they conducted microarray analysis. Figure 1F shows a panel of collagen genes detected in their study in relation to PDAC grade – notably, ITGA1-specific collagens (types 4 and 6) are significantly upregulated in high grade PDAC lesions. In a similar fashion, studies by Garnet *et al*. and Jimeno *et al*. report microarray gene expression data for either tumor cell lines or PDAC patient-derived xenografts, respectively. Figures 1G and 1H demonstrate that ITGA1-specific collagens (types 4 and 6) significantly associate with p53 mutation status and erlotinib resistance, respectively. Together these data suggest a primary role for ITGA1 in mediating pathogenesis in PDAC and that ITGA1 may serve as an early diagnostic biomarker.

### ITGA1 is required for PDAC cell survival and migration

**Figure 2:**
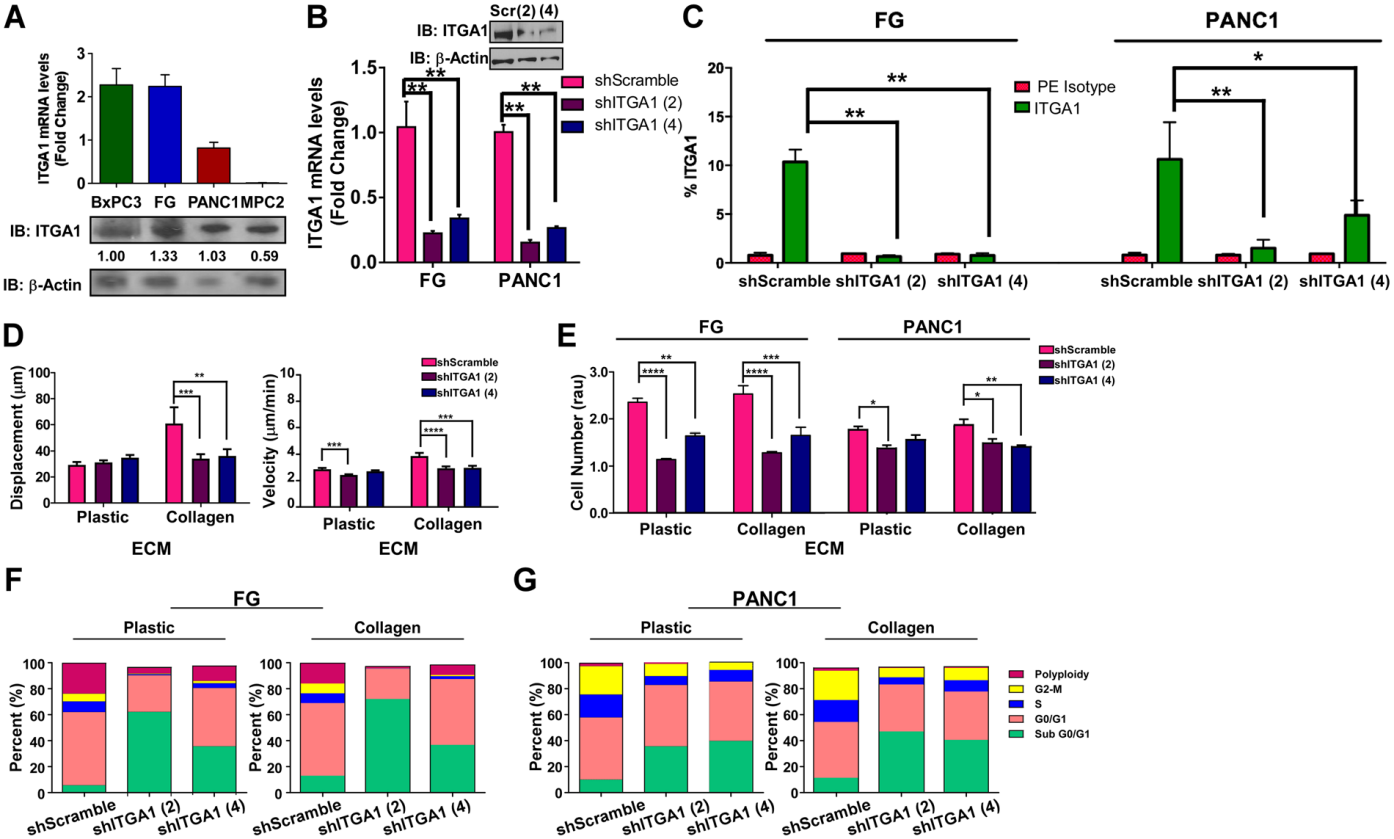
ITGA1 is required for PDAC cell survival and migration. (**A**) qPCR and Western blot analyses of ITGA1 levels in 4 human PDAC cell lines. (**B**) qPCR and Western blot analyses of ITGA1 in virally transduced FG and PANC1 with scramble or two unique ITGA1-specific shRNA constructs – normalized to GAPDH or a-tubulin. (**C**) Flow cytometry analysis of cell surface ITGA1 levels in virally transduced FG and PANC1 cells stained with either IgG1 K Isotype control (red) or Anti-human CD49a (green). Data plotted as percent of cells staining positive of ITGA1. (**D**) Single cell migration assay of transduced PANC1 cells on collagen (3 μg/mL). (**E**) AQeuous One assay was performed on transduced FG and PANC1 cells 72 hours after plating on plastic or collagen. (**F** and **G**) Cell cycle profiles were analyzed 24 hours after plating transduced FG (**F**) or PANC1 (**G**) cells on plastic or collagen. Relative percent of cells in each stage are shown. Figure shows the best representative profile of 2 or more repeats. *, ** and *** indicate t-test derived p-values less than 0.05, 0.01 and 0.001, respectively. Original blot images are cropped to show indicated bands.

We next examined a panel of PDAC cell lines for their expression of ITGA1, finding that the epithelial FG and PANC1 lines expressed the highest levels of ITGA1 among the KRas mutant lines that were tested (Fig. 2A). Using both transient siRNA and stable shRNA expression, we demonstrate successful silencing of ITGA1 in both these lines relative to the appropriate control RNAi constructs (Fig. 2B and Supplemental Figs 2A and C). Importantly, shRNA-mediated knockdown of ITGA1 resulted in loss of protein expression at the cell surface as determined by flow cytometry (Fig. 2C and Supplemental Fig. 2D). ITGA1 loss-of-function studies revealed a critical role for collagen-ITGA1 binding in mediating cell motility (Fig. 2D and Supplemental Videos 1–3). Analysis of cell viability in response to ITGA1 knockdown on plastic and collagen substrates demonstrated that, while ITGA1 is required for cell viability under these conditions, this effect was not substrate specific (Fig. 2E and Supplemental Fig. 2B). To evaluate whether this decrease in cell viability in response to ITGA1 knockdown was due to a shift in the cell cycle profiles, we performed flow cytometry on propidium iodide labeled cells. These data show that the decrease in ITGA1-mediated cell viability correlates with an increase in cell populations containing less than 2n DNA content – those likely undergoing cell death (Fig. 2F,G and Supplemental Fig. 2F). Together, these data support a role for ITGA1 in the metastatic progression of PDAC since ITGA1-mediated cell migration is collagen dependent while ITGA1-mediated cell survival is independent of the extracellular environment.

### ITGA1 is necessary for collagen-induced PDAC cell attachment and spreading

**Figure 3:**
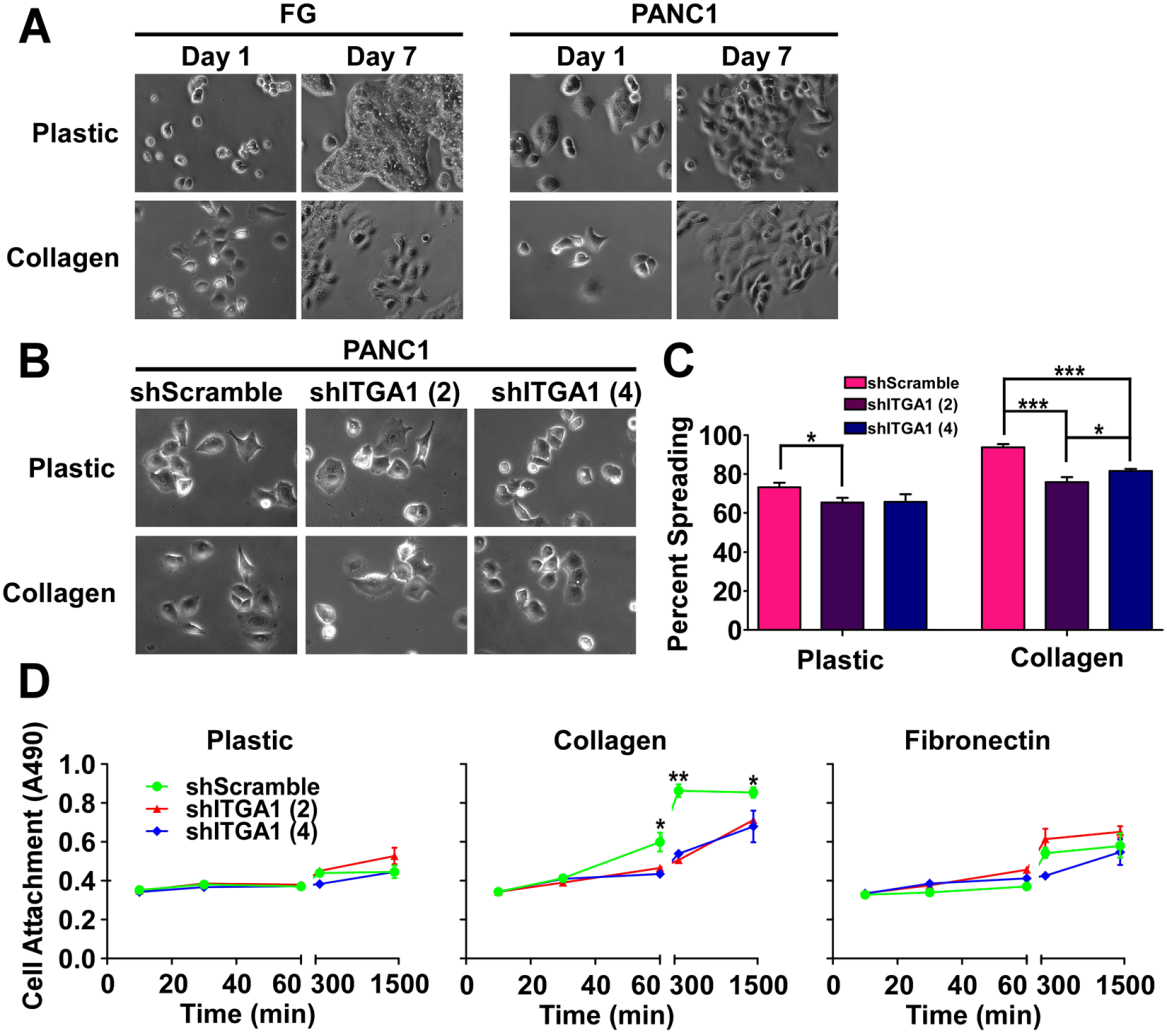
ITGA1 is necessary for collagen-induced PDAC cell attachment and spreading. (**A**) Phase-contrast images of FG and PANC1 cells on plastic or collagen (5 μg/mL) at day 1 and day 7 post-plating. (**B**) Phase-contrast images at day 1 of PANC1 transduced lines plated on plastic or collagen (5 μg/mL). (**C**) Percent spreading quantified from replicates represented in (**B**) of PANC1 transduced lines on day 1 after plating onto plastic or collagen (5 μg/mL). (**D**) A modified cell viability assay was used to detect attachment of PANC1 transduced lines at indicated time-points after plating onto plastic, collagen (5 μg/mL) and fibronectin (5 μg/mL). *, ** and *** indicate t-test derived p-values less than 0.05, 0.01 and 0.001, respectively.

Since mesenchymal cell morphology and gene expression signatures are commonly associated with the metastatic potential of pancreatic cancer cells, we evaluated whether collagen could affect the morphology, spreading and attachment of PDAC cells in an ITGA1-dependent manner. As shown in Fig. 3A, collagen induces cell spreading and a shift toward a more mesenchymal morphology in both FG and PANC1 cells. Importantly, ITGA1 silencing markedly reduces the ability of PDAC cells to spread on collagen (Fig. 3B,C). Finally, since cell spreading depends upon initial cell attachment, and cell attachment of PDAC cells to the collagen-rich fibrotic microenvironment is an established prerequisite for cancer cell metastasis^28, 33^, we tested the role of ITGA1 on the kinetics of PANC1 cell attachment to plastic, collagen and fibronectin substrates. Notably, ITGA1 knockdown completely abrogated collagen-induced cell attachment (Fig. 3D). While cells attached slightly faster and to a larger extent to the fibronectin substrate, this effect did not depend upon ITGA1 expression.

### ITGA1 marks and promotes viability of ALDH1^hi^ PDAC cells

**Figure 4:**
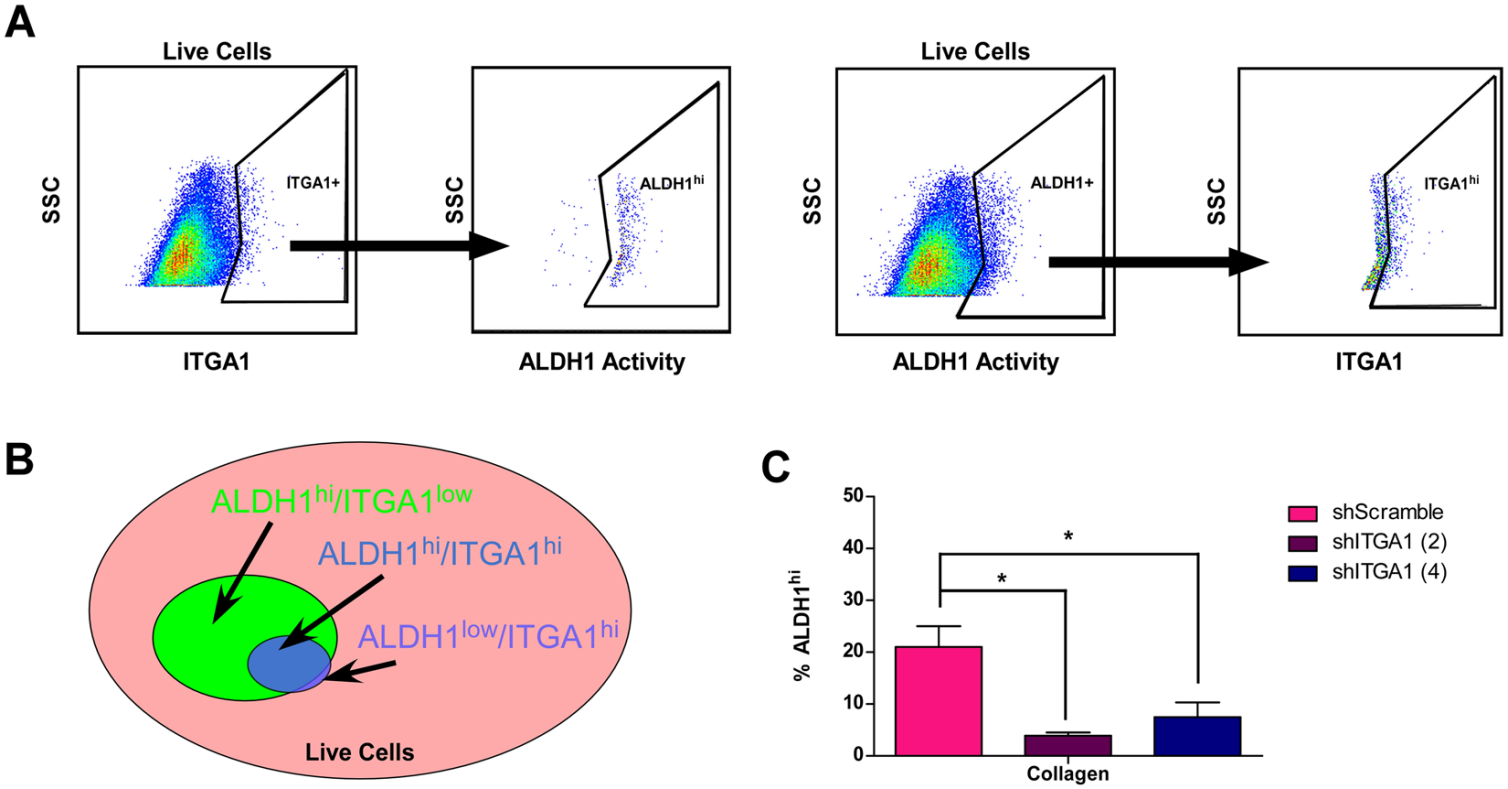
ITGA1 marks and promotes viability of ALDH1hi PDAC cells. (**A**) Live FG and PANC1 cells, stained for cell surface ITGA1 levels using either IgG1 K Isotype control or anti-human CD49a. ALDH1 enzymatic activity in these cells was detected using the ALDEFLUOR assay kit and activity-quenching DEAB reagent as a negative control. (**B**) Venn diagram summarizing the possible relationship between ITGA1 and ALDH1 levels within live PDAC cell population. (**C**) The ALDH1 enzymatic activity was detected using ALDEFLUOR assay for shRNA transduced PANC1 cells 48 hours after plating on Collagen (3 μg/mL). (**C**) *Indicates t-test derived p-value less than 0.05.

Highly tumorigenic stem-like cells were first identified in pancreatic cancer as a subpopulation that labeled positively for CD44, CD24 and EpCAM cell surface antigens^34^. Subsequent work reported that cell surface expression of CD133/CXCR4 marked a more restricted population of metastatic, therapy-resistant tumor-initiating cells^35^. More recently, aldehyde dehydrogenase one (ALDH1) activity has been reported to accurately reproduce CD133 staining in pancreatic cancer as a marker of poor patient prognosis and therapy resistance^36, 37^. We analyzed PANC1 PDAC cells for their dual staining patterns of cell surface ITGA1 and total cellular ALDH1 activity (Supplemental Fig. 2D,E). As reported in Fig. 4A, nearly all cells that stain positive for cell surface levels of ITGA1 are also positive for ALDH1 activity (left). However, only approximately 35% of ALDH1^hi^ cells are also positive for cell surface ITGA1 (Fig. 4A, right). These data are summarized in the Fig. 4B Venn diagram that represents ITGA1^hi^ cells as a subpopulation of ALDH1^hi^ cells. In agreement with these data, depletion of cellular and cell surface ITGA1 levels significantly reduces the viability of ALDH1^hi^ PDAC cells (Fig. 4C).

### ITGA1 is an indicator of EMT and co-expresses with TGFβ response genes in PDAC

Based upon the above data demonstrating a collagen-specific role for ITGA1 in cellular phenotypes involved during local dissemination and systemic spread of pancreatic cancer, we next asked whether ITGA1 co-expressed with markers of EMT. As shown in Supplemental Fig. 3A, ITGA1 positive tumors express lower levels of the epithelial marker E-Cadherin (CDH1) along with elevated levels of select mesenchymal markers (SERPINE1, VIM and ACTA2). TGFβ is a well-established tumor suppressing growth factor; however, loss of SMAD4 function or dysregulated TGFβ signaling can lead to cancer progression and metastasis^38–40^ – often, via the induction of an EMT program^41, 42^. Therefore, we compared TGFβ response genes^16, 17, 43^ against ITGA1 expression levels in multiple pancreatic cancer patient samples using the Cancer BioPortal database. This identified PEAK1, BMPR2, COL4A1 and ZEB1 as ITGA1 co-expressors in pancreatic cancer and suggested that ITGA1 may play a role in regulating TGFβ responses in pancreatic cancer (Supplemental Fig. 3B,C).

### ITGA1 mediates TGFβ/collagen-induced EMT and gemcitabine resistance in PDAC cells

**Figure 5:**
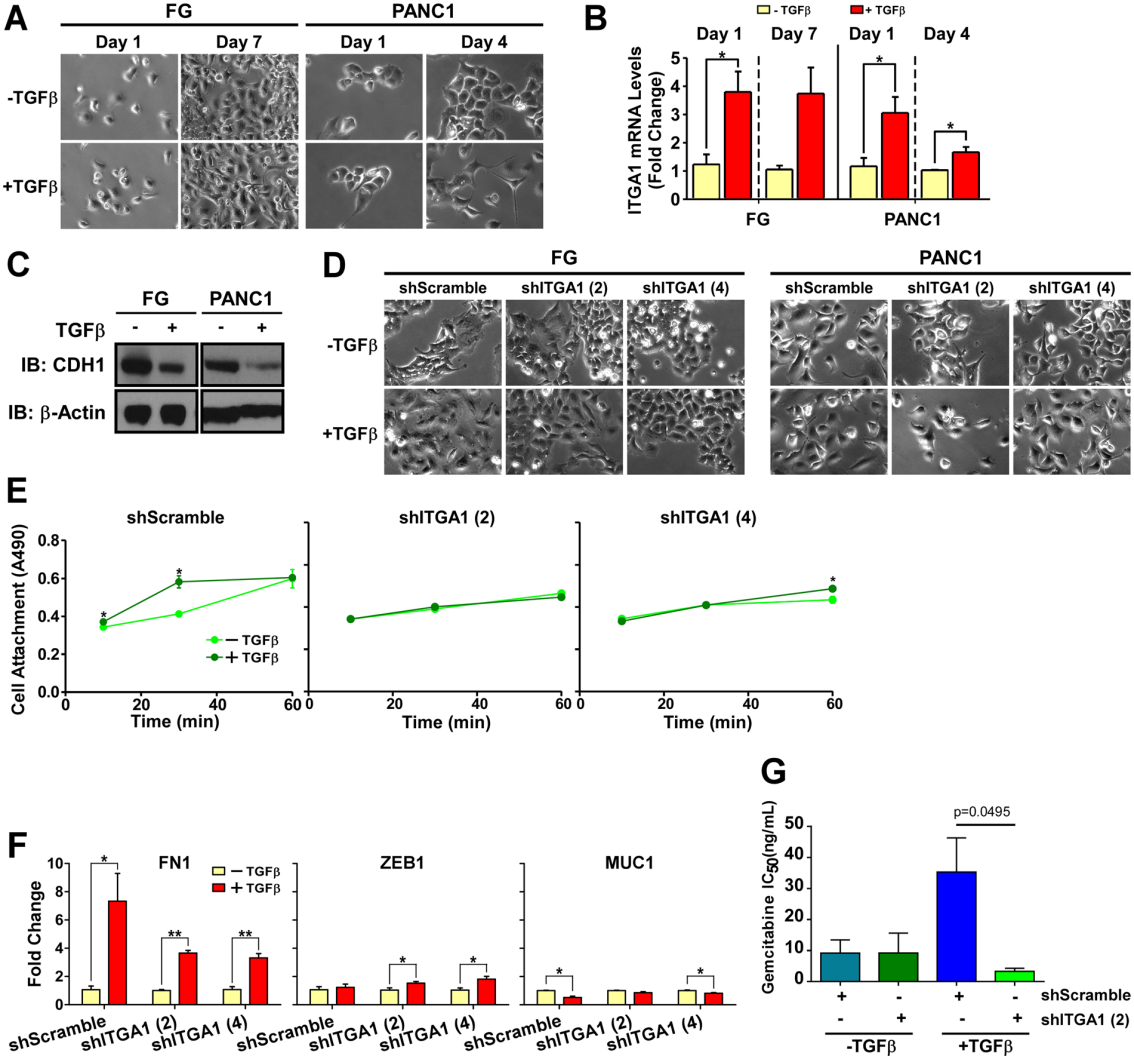
ITGA1 mediates TGFβ/collagen-induced EMT and gemcitabine resistance in PDAC cells. (**A**) Phase-contrast microscopy images of FG and PANC1 cells plated on collagen (5 μg/mL) and treated with TGFβ. (**B**) qPCR for ITGA1 in FG cells – 1 and 7 days post-TGFβ treatment, and in PANC1 – 1 and 4 days post-TGFβ treatment. POLR2A was used as the house-keeping gene. (**C**) Western Blot for CDH1 levels following 7 (FG) or 4 (PANC1) day treatment with TGFβ. Original blot images are cropped to show indicated bands. (**D**) Phase-contrast microscopy of transduced FG and PANC1 lines with or without TGFβ on day 7 and day 4, respectively. (**E**) A modified cell viability assay was used to detect attachment of PANC1 transduced lines with or without TGFβ treatment at indicated time points after plating onto collagen (5 μg/mL). (**F**) qPCR for FN1, MUC1 and ZEB1 expression in transduced PANC1 cells following 4 days of control or TGFβ treatment. (**G**) IC50 values from gemcitabine dose-response curves of FG shRNA cells on collagen (5 μg/mL) and TGFβ treated or untreated. *Indicates t-test derived p-value less than 0.05.

While TGFβ has been previously demonstrated to cooperate with the extracellular matrix to promote the progression of solid tumors^16, 44–46^, surprisingly, this observation has not been made in the context of pancreatic cancer. Here, we report for the first time that TGFβ and collagen cooperate to induce a mesenchymal morphology, downregulate E-Cadherin (CDH1) and upregulate ITGA1 expression in PDAC cells (Fig. 5A–C). While TGFβ can induce a subtle EMT effect in the absence of ECM proteins or in the presence of fibronectin (Supplemental Figs 4A and 5A), ITGA1 levels are not markedly increased and decreased E-Cadherin levels are less pronounced across both cell types under these alternate extracellular substrate conditions (Supplemental Figs 4B,C and 5B,C). We next tested whether ITGA1 expression was necessary for TGFβ/collagen-induced EMT. As shown in Fig. 5D, while ITGA1 knockdown in FG and PANC1 cells did not completely abrogate the mesenchymal morphology shift, ITGA1 depletion caused these cells to retain a more epithelial shape. Importantly, short-term cell attachment of PANC1 cells to collagen was significantly potentiated by TGFβ treatment of the shScramble, but not observed in the ITGA1 knockdown variants of PANC1 cells (Fig. 5E). Notably, this ITGA1-dependent effect was not observed when PDAC cells were treated with TGFβ alone or with TGFβ after cells were plated on fibronectin (Supplemental Figs 4D,E and 5D,E). We next tested the role of ITGA1 expression on TGFβ/collagen-induced FN1, ZEB1 and MUC1 gene expression changes. We found that TGFβ was able to upregulate ZEB1 in the absence of ITGA1 and/or ECM proteins (Fig. 5F and Supplemental Figs 4F and 5F). Furthermore, FN1 increased in response to TGFβ in the presence of collagen or plastic substrates, while only the combination of both TGFβ and collagen was able to reduce MUC1 levels in these cells (Fig. 5F and Supplemental Figs 4F and 5F). Since stem-like mesenchymal cells have been previously reported to resist the cytotoxic effects of gemcitabine (the FDA-approved standard-of-care therapy for PDAC)^35, 47^, we tested whether blockade of ITGA1 sensitizes mesenchymal PDAC cells to gemcitabine. We report here that cooperative TGFβ/collagen signaling decreases gemcitabine potency by nearly 10-fold and that ITGA1 knockdown significantly sensitizes these cells to gemcitabine-induced cytotoxicity (Fig. 5G and Supplemental Fig. 3D), presumably by modulating the mesenchymal-epithelial cell state. Taken together, these data suggest an essential role for ITGA1 in mediating TGFβ signaling crosstalk with the collagen-rich extracellular microenvironment of PDAC during disease progression and therapy resistance.

### ITGA1 is necessary for the metastatic cascade originating from a TGFβ/collagen-rich tumor microenvironment

**Figure 6:**
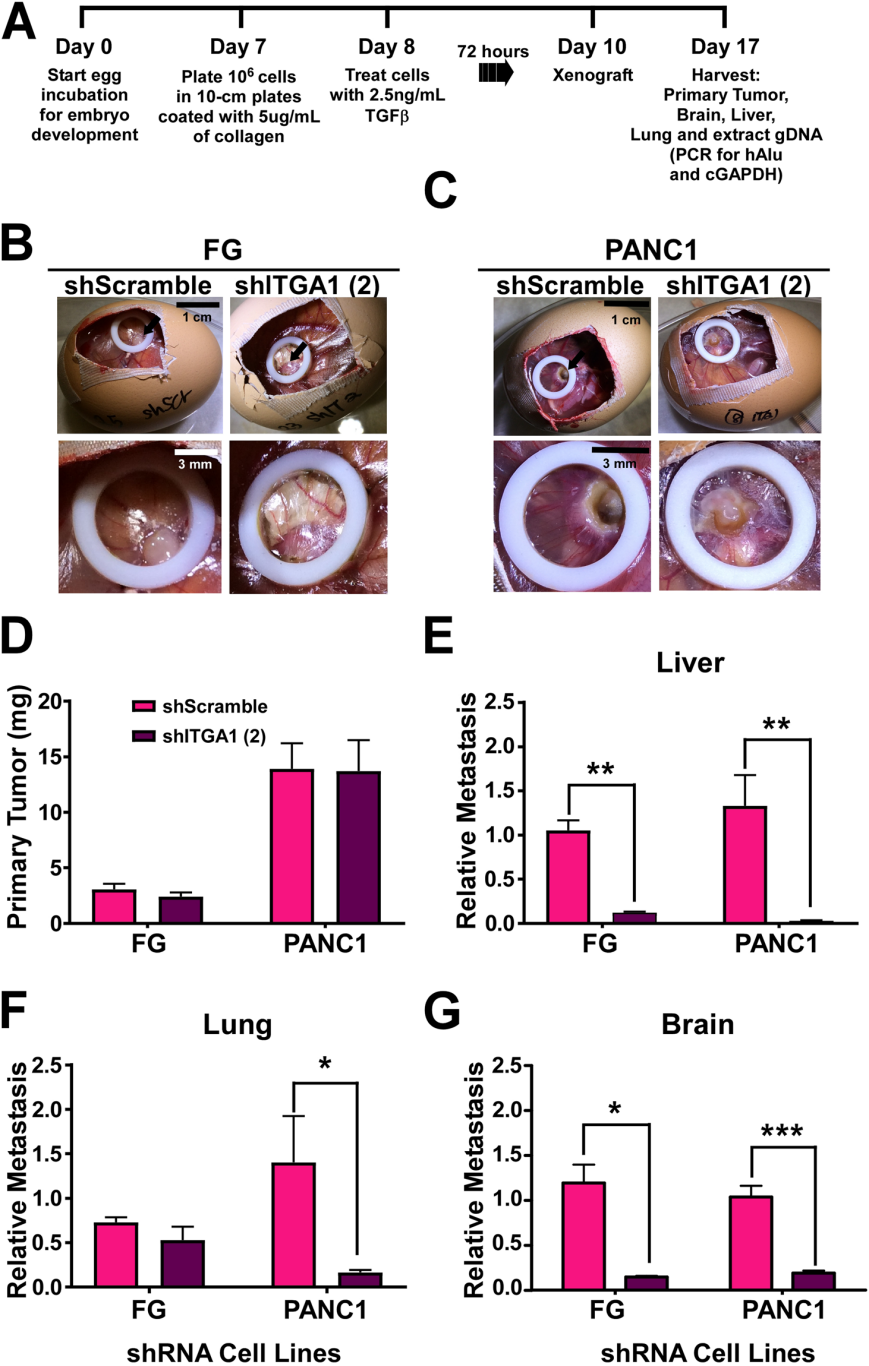
ITGA1 is necessary for the metastatic cascade originating from a TGFβ/collagen-rich tumor microenvironment. (**A**) Experimental scheme to test TGFβ/collagen-induced metastasis of shRNA PDAC cells. (**B** and **C**) Images of the representative CAM tumors for the FG (**B**) and PANC1 (**C**) cells. (**D**) Average primary tumor weight (**E**–**G**) The relative metastases to liver, lung and brain quantified by qPCR detection of human *ALU* repeat genomic DNA against chicken gapdh gDNA. *, ** and *** indicate t-test derived p-values less than 0.05, 0.01 and 0.001, respectively.

To evaluate the role of ITGA1 during PDAC cell metastasis from a tumor microenvironment that is rich in TGFβ and collagen, we employed the chicken embryo chorioallantoic membrane (CAM) metastasis assay. As we and others have previously demonstrated, this system provides the ability to rapidly evaluate organotropism of human tumor cells^16, 19, 48, 49^. Since our *in vitro* data in Fig. 5 demonstrate that ITGA1 is both upregulated and required for long-term EMT changes in PDAC cells, we devised a treatment and xenografting scheme (Fig. 6A) to evaluate the role of ITGA1 on PDAC cell metastasis to the liver, lung and brain. Although, brain metastasis is not common in PDAC patients, we felt it would be relevant to consider the effects of ITGA1 on PDAC cell metastasis to this site as a general indicator of ITGA1 function. Briefly, FG and PANC1 shRNA cell lines were plated onto collagen and pre-treated with TGFβ for 72 hours prior to resuspension and xenografting onto the CAM of 10-day old embryos in the presence of exogenous collagen and TGFβ. Seven days later the primary tumor and other tissues were harvested for analysis. Figures 6B and 6C show sample images of the windowed eggs and tumors on the CAM for the control scramble shRNA and ITGA1-specific shRNA derivatives of the PDAC cell lines. Surprisingly, although our *in vitro* data demonstrate that silencing ITGA1 reduces PDAC cell survival and the frequency of tumor-initiating ALDH1^hi^ PDAC cells, ITGA1 depletion did not affect the *in vivo* average weight of the primary tumors isolated from the CAM tissue (Fig. 6D). However, we observed a marked effect of ITGA1 silencing on the ability for PDAC cells to metastasize to all three tissues – the biggest effect being observed in the liver (a common site of PDAC metastasis) (Fig. 6E–G). Notably, this pro-metastatic effect of ITGA1 was much less apparent in the absence of TGFβ/collagen pre-treatment and co-xenografting (Supplemental Fig. 6), demonstrating a TGFβ/collagen microenvironment-specific role for ITGA1 in pancreatic cancer progression.

## Discussion

Three precursor lesion types have been carefully characterized in the pancreas of patients with and without ductal adenocarcinomas [i.e., intraepithelial neoplasms (PanINs), intraductal papillary mucinous neoplasms (IPMNs) and mucinous cystic neoplasms (MCNs)]. Although histopathologically distinct, they share a very similar genetic basis and progression grading scheme. In particular, PanIN lesions have been shown to precede the onset of ductal adenocarcinoma of the pancreas in a well-defined hierarchical fashion^2, 3, 50^. Given that these PanIN lesions develop within in window of time that affords patients more treatment options which may be curative, methods for the detecting these non-malignant precursor lesions hold great promise^4, 50^. In fact, reports have shown that between 16% to 45% of PanIN-containing pacreata do not have invasive carcinoma^50^, suggesting that these patients can primarily benefit from pre-malignant detection. This urgent need for early detection of these PanIN abnormalities is made even more evident by recent reports showing that systemic dissemination of pancreatic cancer cells occurs even earlier than previously predicted^4^ and before a primary tumor is detectable by histologic analysis^5^. This alarmingly early metastatic progression was further associated with circulating tumor cells that adopted a mesenchymal phenotype and gene expression signature associated with epithelial-mesenchymal transition (EMT) – ultimately, leading to the seeding of distant metastases. In this regard, we report here that ITGA1 is a novel cell surface^26, 51^ and soluble^26, 52^, biomarker for early grade 1 PanIN lesions (Fig. 1C). Moreover, we demonstrate that ITGA1 is frequently upregulated in PDAC patient tissue (Fig. 1A,B), associating with undifferentiated tissue histology and mesenchymal protein expression patterns (Supplemental Fig. 3). Together with our *in vitro* and *in vivo* data demonstrating that ITGA1 expression increases in response to TGFβ/collagen signaling (Fig. 5A–C) and is required for EMT (Fig. 5D–F) and metastasis (Fig. 6), these patient data point to the utility of developing methods for detecting ITGA1 protein or ITGA1-positive cells in serum screening protocols to identify early, invasive, mesenchymal PanIN-derived cell populations in patients^50^.

Previous work has demonstrated that EMT is a critical pre-requisite to the metastatic cascade and that tumor cells commonly readopt epithelial character upon expansion within the metastatic niche^53, 54^. Moreover, recent evidence supports the existence of stem-like mesenchymal cell subpopulations with very high metastatic potential in PDAC^55^. Others have similarly reported that pancreatic tumor-initiating cells with stem-like character can seed pre-metastatic niches and are predominantly positive for the cell-surface CD133 marker^7, 34, 35^. Notably, these tumor-initiating and metastatic cell populations are commonly refractory to gemcitabine, the standard-of-care chemotherapeutic that is FDA-approved to treat PDAC^35^. Interestingly, it has also been reported that tumor cell dormancy within the metastatic niche is driven, in part, by altered ECM-integrin engagement^56, 57^. In agreement with these previous reports, we demonstrate that collagen is capable of modestly reducing the G0/G1 phase in PDAC cells (Fig. 2F,G and Supplemental Fig. 2E). Furthermore, targeting ITGA1 can induce cytotoxicity and reduce their expansion capacity *in vitro* (Fig. 2 and Supplemental Fig. 2). Together with our results demonstrating that ITGA1 inhibition sensitizes PDAC cells to gemcitabine-induced cytotoxicity (Fig. 5G and Supplemental Fig. 3A), ITGA1 represents a key new target for sensitizing both primary and metastatic tumor cells to currently available therapeutic strategies.

Although, EMT has been demonstrated to mark circulating tumor cells from pre-malignant precursor PanIN lesions^5^, recent reports have suggested that EMT may be dispensable for metastasis, driving primarily chemoresistance in pancreatic and breast malignancies^47, 58^. Still, it is clear from our previous work^16^ and the work of others^55, 59–61^ that mesenchymal states within epithelial-derived cancers, such as breast and pancreatic, significantly associate with a higher metastatic potential, worse patient prognosis and therapy refractory nature. Moreover, inducing mesenchymal-epithelial transition (MET) has been proposed as a complimentary therapeutic strategy to improve patient responses to chemotherapy^62^. Thus, EMT is a critical process to understand in the context of pancreatic cancer progression and therapy effectiveness. TGFβ is one of the best characterized inducers of EMT, regulating this process during normal development as well as cancer progression^63–66^. It is also well-accepted that somatic inactivation of TGFβ receptors and Smad proteins are frequent in PDAC, with over half of all patients losing Smad4 function^2, 3^ – often leading to non-canonical signaling pathway activation by TGFβ. Our group and others have reported cooperativity between the ECM protein microenvironment and TGFβ to promote MAPK signaling and tumor progression^16, 17, 67–72^. These studies support previous notions of context-dependent TGFβ responses^40^. In this regard, we demonstrate here that unlike in breast cancer, where TGFβ cooperates with ITGB3-specific ECM proteins, TGFβ preferentially cooperates with ITGA1-specific collagens in PDAC to promote EMT (Fig. 5 and Supplemental Figs 4 and 5), therapy resistance (Fig. 5) and metastasis (Fig. 6). While these data support the development of ITGA1-targeting approaches in cancer tissue, it will be critical to evaluate the systemic effects of this approach or to develop low dose ITGA1-targeting therapies in the context of standard-of-care treatments, as previous work has shown that ITGA1 restricts TGFβ-induced renal fibrosis^73^.

Finally, recent work has suggested that one of the mechanisms by which TGFβ can suppress tumor growth in pancreatic cancer before the loss of Smad4 function is through the induction of a lethal EMT phenotype^41^ - killing nearly all epithelial-like cells. Interestingly, residual mesenchymal-like cells can escape apoptosis and survive under these nutrient restricted *in vitro* and *in vivo* conditions. Notably, the metabolic reprogramming of KRas-driven PDAC has been well characterized and is one key factor leading to disease progression^74^. Data that we present throughout this study demonstrates that under nutrient rich conditions (e.g., as cells invade away from pancreatic PanIN or PDAC lesions and move through circulation) TGFβ can cooperate with collagen ECM proteins via ITGA1-dependent mechanisms to establish viable mesenchymal populations that are required for systemic spread and survival of PDAC tumor cells. Thus, targeting ITGA1 in this context has significant potential to reduce systemic tumor burden and improve patient survival.

## Materials and Methods

### Reagents

Human Fibronectin was purchased from Corning (Cat. # 354008). Human Collagen Type I was purchased from Corning (Cat. #354243). Human transforming growth factor beta was purchased from Peprotech (Cat. #100–21).

### Bioinformatics

#### Oncomine

The top 100 pseudopodium-enriched proteins previously published^15^ were screened for those frequently upregulated in PDAC (as described in Supplemental Fig. 1A) using Oncomine. This yielded 37 prospective genes/proteins enriched in the pseudopodium and upregulated in PDAC.

#### Cytoscape

These 37 genes were used to generate a broad network interactome with the Agilent Literature Search Cytoscape plugin.

#### Babelomics

These same 37 genes were entered into Babelomics and analyzed as a gene set enrichment using the FatiGO tool, generating a hierarchical list of enriched gene ontologies.

### Cell Culture

BxPC3, FG, PANC1 and MiaPaca2 cells were cultured in DMEM/High-Glucose growth media supplemented with 10% FBS, pen/strep and gentamicin. Transduced cells with shRNA were cultured in complete DEME media (as indicated above) with 10 μg/mL puromycin as a selection reagent. Cells were maintained in an incubator at 37 degrees Celsius and 5% CO2.

### Immunohistochemistry

The BIC14011a tissue array slide was purchased from US Biomax. Slides were heated at 60 °C for one hour, incubated in xylene for ten minutes and washed with ethanol several times in five minute increments. Slides were then rinsed with PBS and incubated with endogenous peroxidase quenching solution (3% H2O2 in PBS with 0.3% serum) for ten minutes at room temperature. Slides were then placed in a staining dish with antigen retrieval buffer (0.01 M sodium citrate buffer at pH of 6.0) and boiled under pressure for ten minutes. After cooling with nanopure water rinses, slides were incubated with blocking solution (1.5% goat serum in PBS) inside a humidity chamber for twenty minutes. ITGA1 primary antibody (Abcam, 1.0 μg/mL – 1:500 dilution) was prepared in blocking serum and incubated on the slide at 4 °C humidity chamber overnight. Slides were rinsed with PBS twice and incubated with biotinylated secondary anti-rabbit IgG antibody solution prepared in blocking solution (Vector Laboratories, 1:200 dilution) for one hour at room temperature in a humidity chamber. Slides were washed with PBS once and incubated with Vectastain ABC reagent for thirty minutes. One PBS wash was performed prior to treating with ImmPACT NovaRED peroxidiase substrate solution at room temperature for approximately five minutes. After desired staining was achieved, the slide was washed with nanopure water and counterstained with Hematoxylin QS for approximately eight minutes. Final ethanol and xylene washes were conducted before covering the slide with Permount and coverslip for imaging.

### Quantitative PCR

RNA was extracted from whole cells using the Thermo Scientific GeneJet RNA purification kit following the instructions provided. NanoDrop was used to determine the respective RNA concentrations as well as purity. The Thermo Scientific cDNA synthesis kit was used to synthesize cDNA using 100 ng of template, Maxima Enzyme Mix and 5X Reaction Mix. The samples were run on a thermocycler according to the kit specifications. NanoDrop was used to determine the respective cDNA concentrations as well as purity. The cDNA samples were diluted to 22.5 ng/mL to perform qPCR. Primers were purchased from Integrated DNA technologies and used at a concentration of 10 nmol/mL. 8.75 μL of nuclease-free water was mixed with 2.5 μL of diluted cDNA, 1.25 μL of gene-specific primer and 12.5 μL of Thermo Scientific Maxima SYBR Green. Samples were run on an ABI7300 instrument.

### Western Blot

Cells were lysed with Stringent RIPA Buffer and rotated at 4 degrees Celsius for ~3–4 hours. Lysates were precleared by centrifugation and the protein concentration was determined via a Bradford Assay. 4–12% Bis-Tris gels were ran and transferred to nitrocellulose membranes. The membranes were probed at 4 degrees Celsius overnight with indicated antibodies with the following dilutions: ITGA1 (abcam 1:1000), E-cadherin (cell signaling 1:1000), PEAK1 (milipore 1:1000), GAPDH (1:1000). Secondary antibodies were used at 1:10,000 dilutions. Band intensities were quantified using Fiji software after image thresholding. Pixel intensity was collected from boxes of the same size placed around different bands – protein of interest: housekeeping protein ratios were calculated and plotted.

### RNAi

#### siRNA Transfection

5 × 10^4^ cells were plated in 500 μL of antibiotic-free media in a 24-well plate then incubated in 37 C for 24 hours. After 24 hours, prepared a 5 μM siRNA solution in 1X siRNA buffer. For each sample, 2.5 μL of 5 uM siRNA solution and 47.5 μL of antibiotic/serum-free media was prepared in an Eppendorf tube and incubated for 5 minutes at room temperature. During the 5-minute incubation time for the siRNA solution, prepared a DharmaFECT2 solution that is enough for 3.5 samples with 4.67 μL of DharmaFECT2 reagent and 170.33 μL of antibiotic/serum-free media and incubated this solution for 5 minutes in room temperature. After 5-minute incubation of both solutions, mixed 50 μL of DharmaFECT2 solution with each of the (50 μL) siRNA solution resulting in 100 μL/sample. Incubate this in room temperature for 20 minutes. After incubation, add 400 μL of antibiotic-free complete (FBS) media to each of the Eppendorf tubes containing both the siRNA and DharmaFECT2 solution, mix well. Replace the media within the 24-well plate with the new transfection mix (500 μL/well) and replace the untransfected wells with 500 μL of antibiotic-free complete media. 24-hours after cells are treated with the transfection mix, replace wells with 500 μL of antibiotic-free complete media. 48-hours post transfection, harvest either lysates or protein for quantification.

#### Lentiviral Transduction

FG and PANC1 cells were plated at 1.6 × 10^4 cells/well into a 96-well plate and left to attach overnight. Cells were then treated with 10 μL of viral particles containing a puromycin resistant pKLO.1 vector with a scramble shRNA, ITGA1-specific shRNA (3′-UTR targeting) in 110 uL of complete media and left to incubate for 18 hours, after which the media was changed. The following day, media was changed and supplemented with 10 μg/mL puromycin. Media was changed with puromycin supplemented media every 3 days until resistance was definite. Cells were then maintained in media containing puromycin.

### Cell Viability Assay

24-well plate was coated for one hour with 3 ug/mL of collagen. Cells were plated at 1 × 10^4^ cell/mL in 200 μL and placed in an incubator for 72 hours. 72 hours post plating, 40 μL of AQeuous One solution was added to each well and the absorbance was taken at 1.5, 2, and 3-hour post-addition of the AQeuous One Solution.

### Cell Motility

24-well plate was coated with collagen (3 μg/mL) in 1 mL and incubated for 1 hour at 37 C. After one-hour incubation, the wells were washed with 1 mL of media twice. Then, cells were plated at 1 × 10^4^ cells/mL in 1 mL per well. The plated cells were incubated at 37 C over night (minimum of 16 hours). The next day, media was changed with new media with HEPES buffer solution 1X. Prior to imaging, each wells were filled with media and properly greased to exclude any bubbles to float in the wells when the lid is sealed. We selected minimum of three points per well and imaged for 24 hours post greasing using LEICA microscope. After 24 hours of imaging, cell velocity and displacement were quantified using the Fiji software.

### Cell Morphology and Spreading Assay

6-well plates were either uncoated (plastic) or coated with collagen (5 μg/mL). PANC1-transduced cells were plated at 5 × 10^4^ cells/mL in 2 mL per well. Cells were imaged at six different positions per well at 10X magnification using phase-contrast microscopy 24 hours post-plating. All cells from each position were analyzed and were classified as spread or unspread – cells were counted as spread when lamelapodia or pseudopodia were observable on a given cell. Percent spreading was calculated from the number of cells spread relative to the total number of cells per position.

### Cell Attachment Assay

96-well plates were either uncoated (plastic) or coated with collagen (5 μg/mL) or fibronectin (5 μg/mL). PANC1-transduced cells pretreated for 48 hours with 2.5 ng/mL TGFbeta or 0.1% BSA were plated at 1.0 × 10^4^ cells/well in 200 mL serum-free media per well. Wells were aspirated, washed and refilled at the time points of 10 min, 30 min, 60 min, 6 hrs and 24 hrs. After the wells were refilled, 40 μL of AQueous One reagent was added to each well and incubated at 37 °C for 3 hours. Absorbance readings were measured at 490 nm. Student t-test analysis was used to determine statistical significance.

### Fluorescence Activated Cell Sorting

#### Cell Cycle Analysis

6-well plate was coated with collagen (3 μg/mL) in 2 mL and incubated for 1 hour at 37 °C. After one-hour incubation, the wells were washed with 1 mL of media twice. Then, cells were plated with concentration of 1 × 10^5^ cells/mL in 2 mL per well. The plated cells were incubated at 37 °C over night (minimum of 16 hours). The next day, the cells were removed from the wells with 1 mL/well of 0.25% trypsin. These cells were pelleted at 1000 rpm for 5 minutes. Then, we resuspend the pellets in 1 mL of of PBS and add 2.5 mL absolute ethanol to each sample – incubated on ice for 15 minute. During the 15-minute incubation, we prepared a PI solution (505.25 μL/sample) for staining which included: 475 μL of PBS, 25 υL of 1 mg/mL Propidium Iodide, 5 υL of 10 mg/mL RNase A, and 0.25 υL of 0.05% Triton X-100. After the 15-minute ethanol incubation, the samples were pelleted at 1500 rpm for 5 minutes and resuspended in 500 υL/sample PI solution. The resuspended samples are incubated at 37 °C for 40 minutes. After 40 minutes, we added 3 mL of PBS to each samples and centrifuged at 1500 rpm for 5 minutes. Then, we resuspended the pellets in 500 μL of PBS. We used the FL-2 channel (546 nm) on a BD Facs Calibur instrument to characterize the sample’s cell cycle profile.

#### ALDEFLUOR Assay

10-cm plates were coated with collagen (3 μg/mL) in 5 mL 72 hours before harvesting. 1 × 10^6^ cells were collected per sample in 1 mL of medium and centrifuged at 1000 rpm for 5 minutes. They were resuspended in 1 mL of ALDEFLUOR assay buffer that was provided with the ALDEFLUOR kit. Each sample were split into two tubes (500 μL/tube) and each received 2.5 μL of Activated ALDEFLUOR reagent which was prepared according to the manual’s instruction. Immediately after, one of the tubes also received 5 μL of DEAB reagent to inhibit ALDH1 activity. These cells were incubated in 37 °C for 45 minutes before flow analysis using the FACS machine.

#### Cell Surface ITGA1 (CD49a) Staining Assay

10-cm plates were coated with collagen (3 μg/mL) in 5 mL 72 hours before harvesting. 1 × 10^6^ cells were collected per sample in 1 mL of medium and centrifuged at 1000 rpm for 5 minutes. They were resuspended in 1 mL of PBS. Each sample were split into two tubes (500 μL/tube) and each received 10 μL of PE Isotype IgG1 K Isotype Control reagent or 10 μL of PE Mouse Anti-Human CD49a reagent. These cells were incubated in 37 °C for 45 minutes before flow analysis using the FACS machine.

#### Dual Staining Assay

Cells were harvested directly from a 10-cm plate using PBS and 0.25% trypsin. After pelleting the cells at 1000 rpm for 5 minutes, these cells were resuspended in 5 mL of 10% FBS medium. 2 × 10^6^ cells were aliquoted and spun at 1000 rpm for 5 minutes. After aspirating the supernatant, the tubes with the pellets were immediately transferred outside of the hood onto ice. Resuspended the pellet in 2 mL of ALDEFLUOR assay buffer then split into two tubes (1 mL/tube) and labeled as PE or CD49a. To both tubes, added 5 μL of activated ALDEFLUOR reagent and were split into two tubes (0.5 mL/tube) and labeled as follows: PE-DEAB, PE-Test, CD49a-DEAB, or CD49a-Test. Immediately after, added 5 μL of ALDEFLUOR DEAB reagent to the tubes labeled DEAB. To the tubes labeled PE, added 10 μL of PE Mouse IgG1 K Isotype Control reagent and to the tubes labeled with CD49a, added 10 μL of PE Mouse Anti-Human CD49a reagent. All tubes were wrapped in foil and incubated at 37 °C for 45 minutes. After 45 minutes, the tubes were centrifuged at 1000 rpm for 5 minutes and pelleted in 0.5 mL of ALDEFLUOR assay buffer. These samples were placed on ice until flow cytometry was performed.

### TGFβ-Induced EMT

Cells were plated at 5 × 10^4^ cells/mL in 2 mL in a 6-well plate and left to attach overnight. Cells were then treated with 2.5 ng/mL TGFβ or 0.1% BSA. Cells were maintained (media changed every 48 hours and passaged when necessary) and retreated every 48 hours. Cells were expanded as needed. Cells were either lysed with RIPA Lysis Buffer containing phophotase and protease inhibitors and a western blot was performed or RNA was harvested using the Thermo Scientific GeneJet RNA purification kit.

### Gemcitabine Dose-Response Curves

shRNA-transduced FG cells were cultured on tissue culture plastic or collagen at 3 μg/mL for 7 days in the presence or absence of TGFβ at 2.5 ng/mL. Cells were then seeded as described in the AQueous One Assay procedure and treated with a dose range of gemcitabine across 3 orders of magnitude including the previously established IC50 for these cells. The assay was harvested 96 hours later and dose-response curves were generated using Graph Pad Prism.

### Chorioallantoic Membrane (CAM) Metastasis

Chicken eggs were purchased from Meyer Hatchery, and incubated for 10 days at 37 °C, 60% humidity. The CAM Assay was preformed according to a previously described protocol^48^. On day 10 post fertilization, the CAM was dropped and the eggs were windowed. A plastic ring was then placed on the CAM and cells were xenografted within the ring. Surgical tape was then placed over the window, and the egg was then placed back into the incubator. Alternatively, cells were prepared as described in Fig. 6A prior to xenografting. After an additional 7 day incubation time, the egg was opened and the primary tumor was removed from the CAM. The embryo was then extracted from the egg and sacrificed. Liver, lung and brain tissue was collected and flash frozen then stored at −80 °C until processed. Upon processing, the tissue was thawed on ice, weighed and homogenized in digestion solution. Samples were then heated at 57 °C for 5 hours. Genomic DNA was then extracted using Thermo Scientific GeneJet Genomic DNA purification kit. DNA concentration was then quantified by NanoDrop. gDNA was then diluted to 22.5 ng/mL and qPCR was preformed for human *alu* and chicken gapdh sequences. Relative metastasis was calculated as previously described^48^. Briefly, dCt values were calculated as alu_Ct_ - gapdh_Ct_. ddCt values were calculated as dCt_shITGA1_ -Average dCt_shScramble_. RQ values were calculated as 2^e(−ddCt)^.

### Statistical Significance Analysis

Unless noted otherwise in figure legends, statistical significance was calculated as unpaired p-value of less than 0.05 via Student’s T-test using GraphPad Prism (GraphPad Software Inc.). All graphs show representative mean + SEM from across at least three experimental replicates.

### Data Availability

The datasets generated during and/or analysed during the current study are available in the Oncomine (www.oncomine.org), Human Protein Atlas (http://www.proteinatlas.org) and Cytoscape Agilent Literature Search (http://www.cytoscape.org/) repositories.

## Electronic supplementary material

Supplemental Information

Supplemental Data Table

Supplemental Movie 1

Supplemental Movie 2

Supplemental Movie 3
