# Supplementary material for: ITGA1 is a pre-malignant biomarker that promotes therapy resistance and metastatic potential in pancreatic cancer
Source: Sci Rep. 2017 Aug 30;7:10060. doi: 10.1038/s41598-017-09946-z (PMC5577248; doi:10.1038/s41598-017-09946-z)
Supplement: Supplementary file 1 — Supplemental Information [file 41598_2017_9946_MOESM1_ESM.pdf]

ITGA1 is a pre-malignant biomarker that promotes therapy resistance and metastatic potential in pancreatic cancer

Armen Gharibi[1‡], Sa La Kim[1‡], Justin Molnar[1], Daniel Brambilla[1], Yvess Adamian[1], Malachia Hoover[1], Julie Hong[1], Joy Lin[1], Laurelin Wolfenden[1], Jonathan A. Kelber[1*]

[1]Department of Biology, California State Univeristy Northridge
[‡]These authors contributed equally to this work.
[*]Correspondence addressed to jonathan.kelber@csun.edu

SUPPLEMENTAL INFORMATION:

Supplemental Figures 1-6

Supplemental Table 1

Supplemental Movies 1-3

Supplemental Figure 1

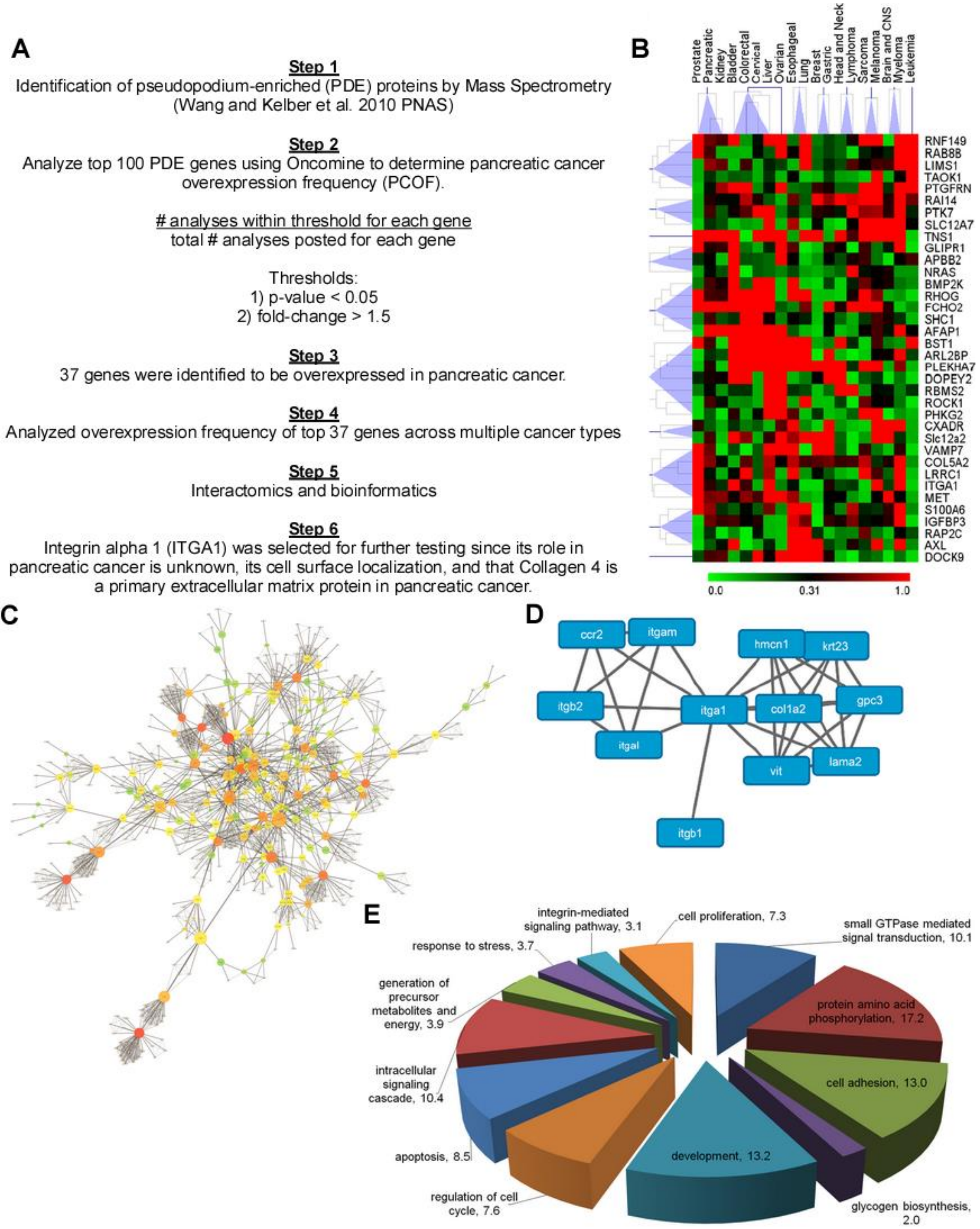

Supplemental Figure 1: (A) Flow chart illustrating the steps taken to identify ITGA1 as a potential biomarker in PDAC. (B) Heat map of 37 PCOR genes and their overexpression frequency in PDAC and other malignancies. (C) Interactome of the top 37 PDE proteins. (D) Sub-interactome for ITGA1. (E) Pie-chart illustrating the molecular function and/or biological process gene ontologies of the 37 genes shown in panel (B).

Supplemental Figure 2

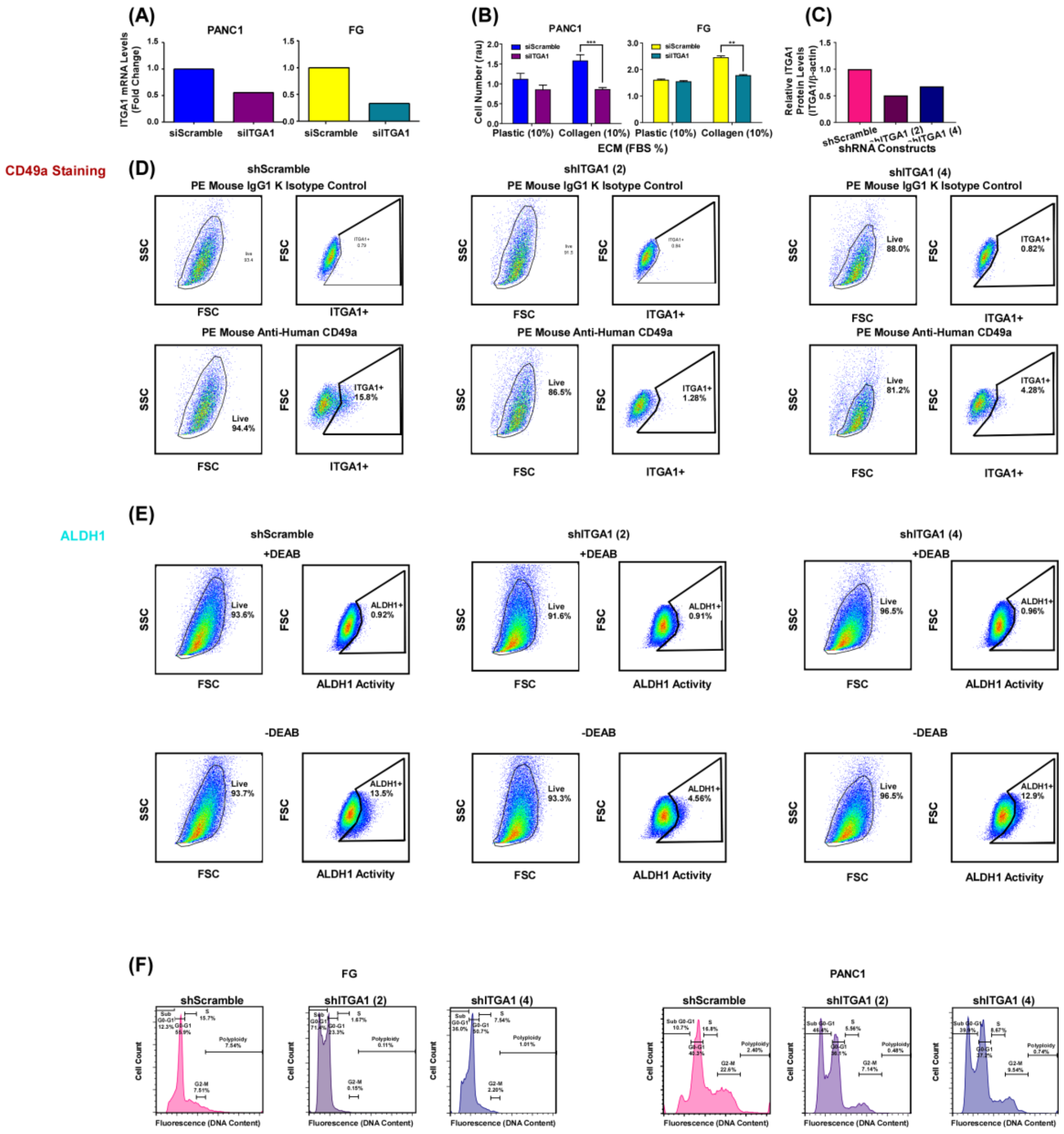

Supplemental Figure 2: (A) qPCR analyses of ITGA1 in siRNA-transfected FG and PANC1 normalized to either GAPDH or alpha-tubulin. (B) AQeuous One Assay was performed on transfected FG and PANC1 cells 72 hours after plating on plastic or collagen. (C) Quantification of total lysate ITGA1 levels in the shRNA lines shown in Figure 2B inlay. (D-F) Gating strategy used during Flowjo analysis. (D) Cell surface levels of ITGA1 were quantified by staining for either IgG1 K Isotype control or anti-human CD49a. The forward and side scatter (FSC vs SSC) gating strategy was utilized to exclude debris from subsequent analyses. ITGA1-positive cells were identified through FL2-H channel in the IgG1 K Isotype control samples first and the gates were applied to anti-human CD49a samples. (E) ALDH1-high cells were identified through FL1-H channel in DEAB+/Aldefluor+ samples and the gates were applied to DEAB-/Aldefluor+ samples. (F) Cell cycle stages were quantified by measuring the DNA content of cells that were stained with Propidium Iodide in FL2-H channel.

Supplemental Figure 3

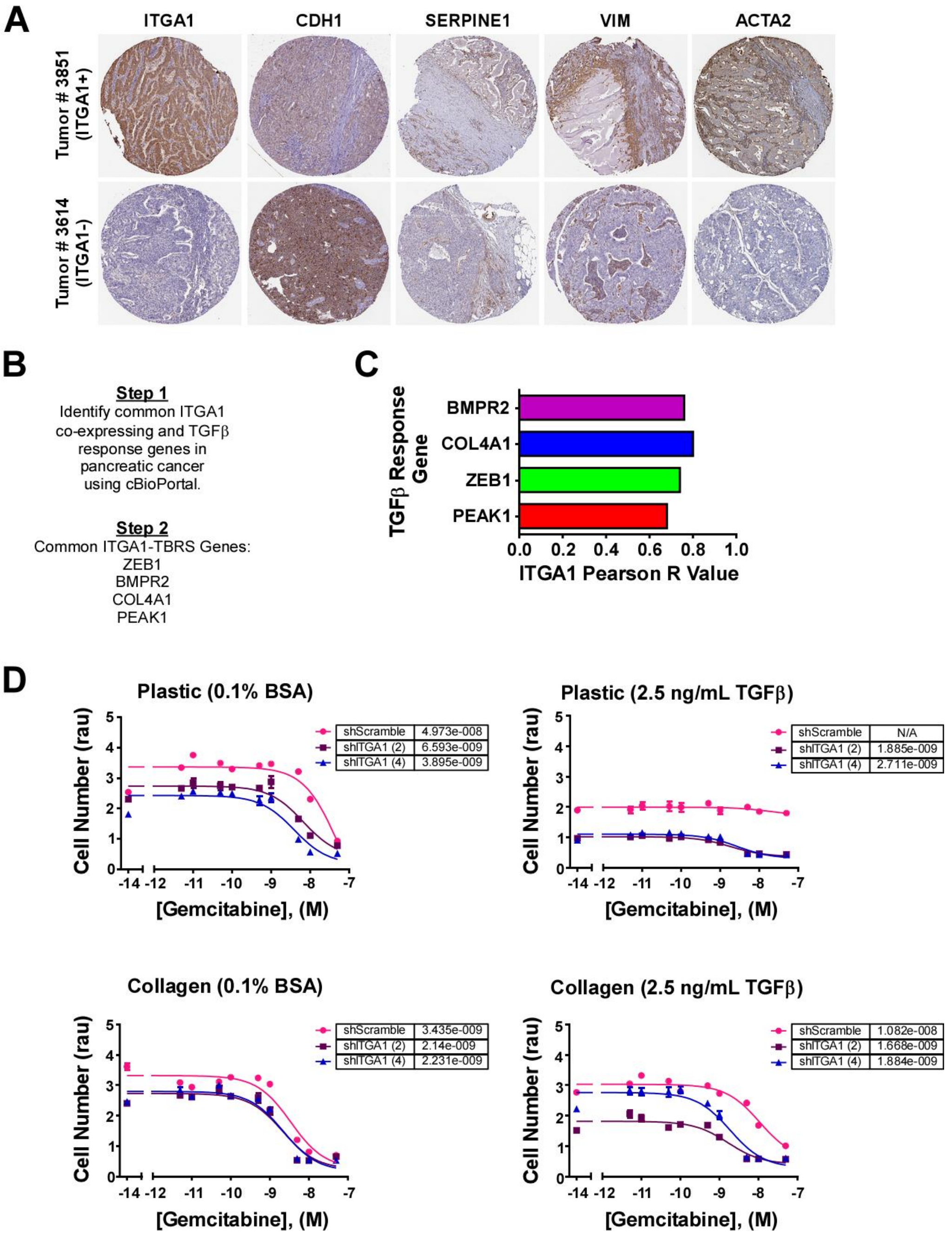

Supplemental Figure 3: (A) Immunohistochemistry staining for ITGA1 and EMT-markers in ITGA1+ and ITGA1- PDAC patient tissue. (B) Flow chart illustrating the steps taken to identify ITGA1 co-expressors that are also TGFβ response genes. (C) Cancer BioPortal co-expression Pearson R-values of ITGA1 and common TGFβ response genes in pancreatic cancer. (D) Dose response curves for gemcitabine-induced cytotoxicity on FG shRNA cells pre-treated with 2.5 ng/mL TGFβ.

Supplemental Figure 4

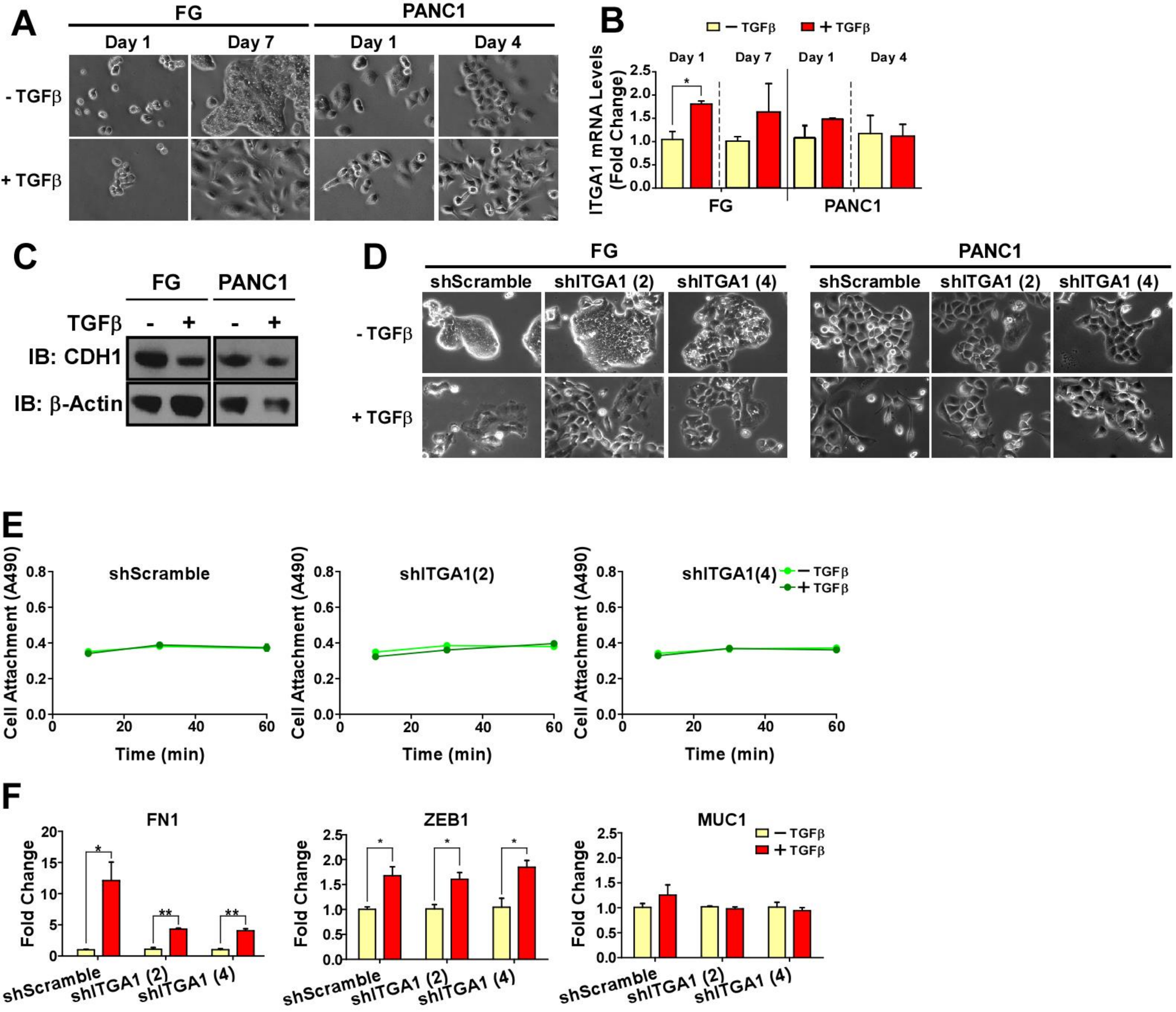

Supplemental Figure 4: (A) Phase-contrast microscopy images of FG and PANC1 cells treated with TGFβ plated on plastic. (B) qPCR for ITGA1 in FG cells – 1st and 7th days post-TGFβ treatment and in PANC1 – 1st and 4th day post-TGFβ treatment. POLR2A was used as the house-keeping gene. (C) Western Blot for CDH1 levels following 7 (FG) or 4 (PANC1) day treatment with TGFβ. Original blot images are cropped to show indicated bands. (D) Phase-contrast microscopy of transduced FG and PANC1 lines with or without TGFβ on day 7 and day 4, respectively. (E) Cell viability assay using AQueous One was performed on PANC1 transduced lines with or without TGFb treatment for indicated time-points. (F) qPCR for FN1, MUC1 and ZEB1 expression in transduced PANC1 cells following 4 days of control or TGFβ treatment. * and ** indicates t-test derived p-values less than 0.05 and 0.01, respectively. All TGFβ treatments were 2.5 ng/mL.

Supplemental Figure 5

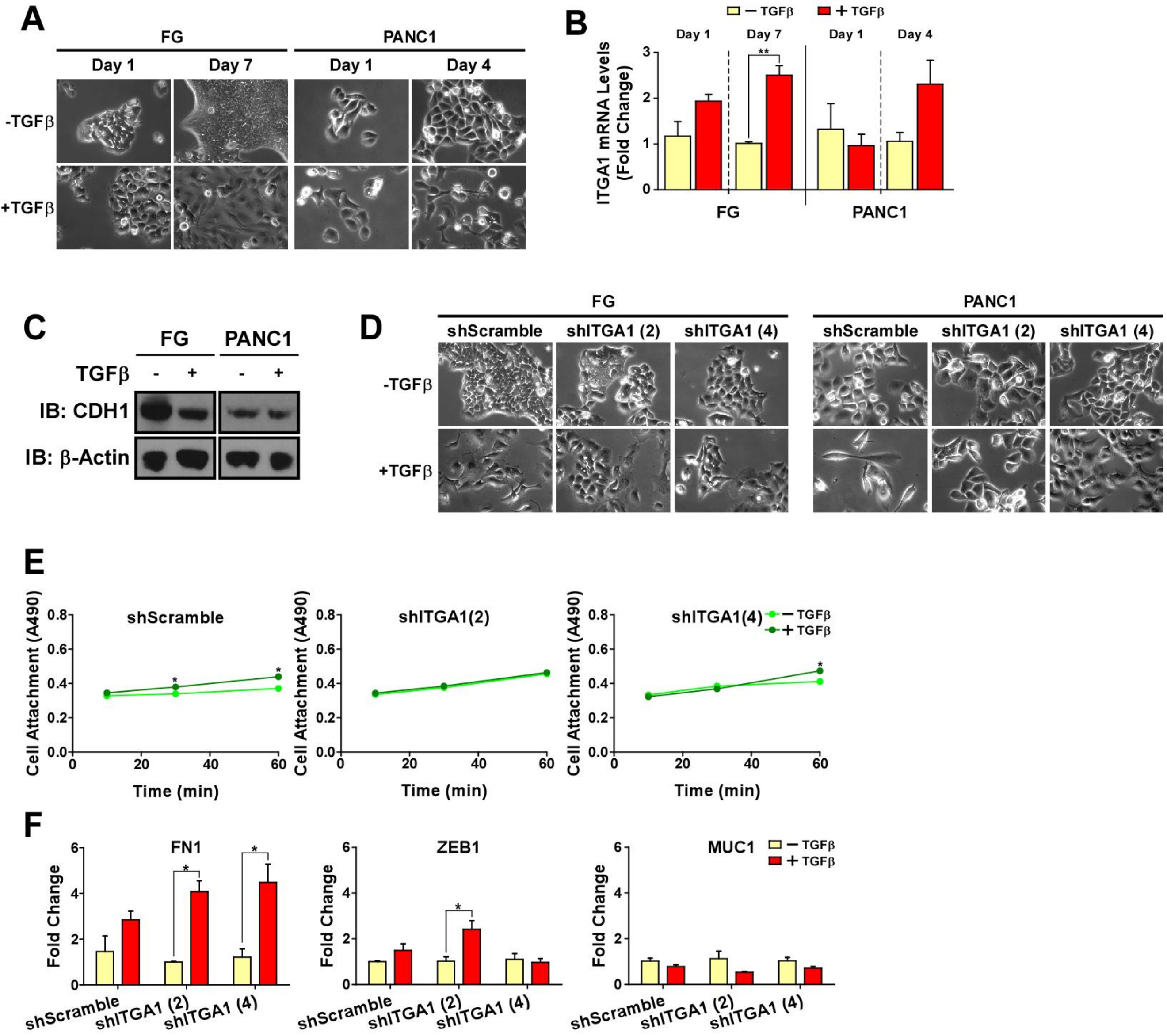

Supplemental Figure 5: (A) Phase-contrast microscopy images of FG and PANC1 cells treated with TGFβ plated on fibronectin (5μg/mL). (B) qPCR for ITGA1 in FG cells – 1st and 7th days post-TGFβ treatment and in PANC1 – 1st and 4th day post-TGFβ treatment. POLR2A was used as the house-keeping gene. (C) Western Blot for CDH1 levels following 7 (FG) or 4 (PANC1) day treatment with TGFβ. Original blot images are cropped to show indicated bands. (D) Phase-contrast microscopy of transduced FG and PANC1 lines with or without TGFβ on day 7 and day 4, respectively. (E) Cell viability assay using AQueous One was performed on PANC1 transduced lines with or without TGFβ treatment for indicated time-points. (F) qPCR for FN1, MUC1 and ZEB1 expression in transduced PANC1 cells following 4 days of control or TGFβ treatment. * and ** indicates t-test derived p-values less than 0.05 and 0.01, respectively. All TGFβ treatments were 2.5 ng/mL.

Supplemental Figure 6

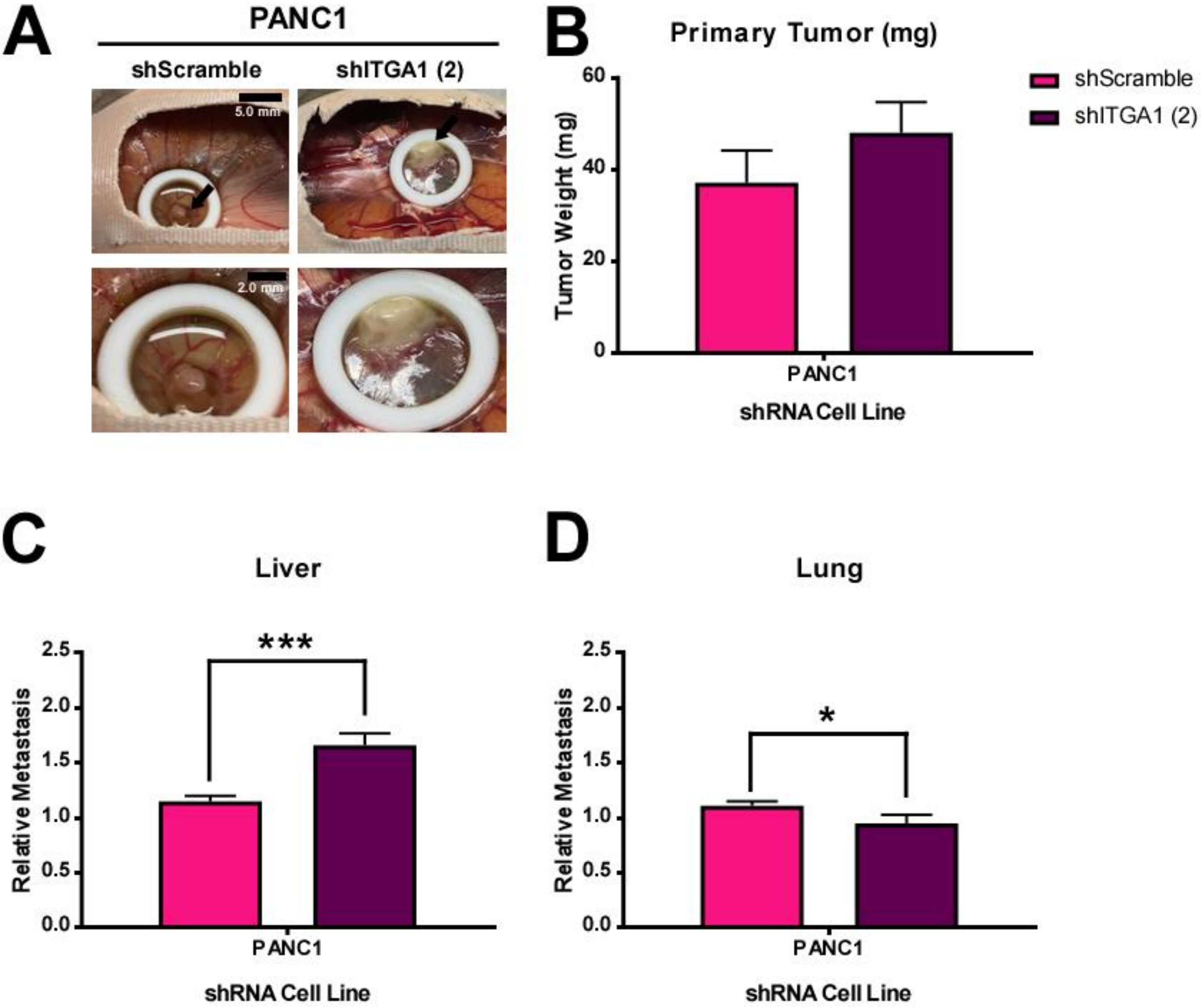

Supplemental Figure 6: (A) Images of the primary tumor on CAM (indicated by arrows) and (B) corresponding tumor weight (mg). (C-D) The relative metastases to liver, and lung were quantified by detecting human ALU repeat genomic DNA against chicken gapdh DNA via qPCR. * and ** indicates t-test derived p-values less than 0.05 and 0.01, respectively.
